# Dynamic Surface Properties of α-Lactalbumin Fibril Dispersions

**DOI:** 10.3390/polym15193970

**Published:** 2023-10-02

**Authors:** Boris Noskov, Giuseppe Loglio, Reinhard Miller, Olga Milyaeva, Maria Panaeva, Alexey Bykov

**Affiliations:** 1 Institute of Chemistry, St. Petersburg State University, Universitetsky pr. 26, St. Petersburg 198504, Russia; b.noskov@spbu.ru (B.N.);; 2 Institute of Condensed Matter Chemistry and Technologies for Energy, 16149 Genoa, Italy; 3 Department of Physics, Technical University of Darmstadt, 64289 Darmstadt, Germany

**Keywords:** α-lactalbumin, amyloid fibrils, surface dilational elasticity, surface pressure, molten globules, stability of fibril dispersions

## Abstract

The dynamic surface properties of aqueous dispersions of α-lactalbumin (ALA) amyloid fibrils differ noticeably from the properties of the fibril dispersions of other globular proteins. As a result, the protocol of the application of ALA fibrils to form stable foams and emulsions has to be deviate from that of other protein fibrils. Unlike the fibrils of β-lactoglobulin and lysozyme, ALA fibrils can be easily purified from hydrolyzed peptides and native protein molecules. The application of the oscillating barrier method shows that the dynamic surface elasticity of ALA fibril dispersions exceeds the surface elasticity of native protein solutions at pH 2. ALA fibrils proved to be stable at this pH, but the stability breaks at higher pH levels when the fibrils start to release small peptides of high surface activity. As a result, the dynamic surface properties of ALA coincide with those of native protein solutions. The ionic strength strongly influences the adsorption kinetics of both fibril dispersions and native protein solutions but have almost no impact on the structure of the adsorption layers.

## 1. Introduction

The interest in amyloid fibrils, which can be formed by various proteins, has increased significantly over the last ten years after it was discovered that they were not always toxic but had important functions in living organisms and could have numerous applications due to their properties; in particular, they can be used for drug delivery, solar energy conversion, water purification, 3D printing, biosensing and cell differentiation [1,2,3,4,5,6,7]. Some of the applications are connected with the high surface activity of protein fibrils [8], particularly with their ability to stabilize foams and emulsions in the food industry [3,5,9,10,11,12,13,14,15,16,17,18,19,20,21,22,23]. Although it is now recognized that protein fibrils can be effective stabilizers of disperse systems [10,11,15,16,23], the stabilization mechanism is not sufficiently clear [16,23]. One of the problems consists of the determination of the surface properties of the dispersions of pure protein fibrils. The fibril formation at elevated temperatures usually results in a mixture of fibrils, native proteins, and peptides of relatively low molecular weight, which determine the kinetic dependencies of surface properties [13,16,23,24,25]. The elimination of the influence of contaminations and estimation of the surface properties of aqueous dispersions of pure protein fibrils is not a simple task. At the same time, it has been shown recently that the influence of impurities becomes much weaker if the protein aggregates at the liquid–gas interface are not adsorbed from the bulk phase but spread directly onto the surface from a concentrated dispersion [25,26]. In this case, the local surface concentration of impurities is close to that in the bulk phase and is much lower than in the case of aggregate adsorption layers where the small peptides are accumulated at the interface due to their high surface activity and fast adsorption. As a result, the properties of spread layers at the liquid surface are determined mainly by the fibrils.

Practically all published experimental results on the properties of adsorption layers of aggregates of animal proteins relate to β-lactoglobulin (BLG) fibrils and spherulites or the aggregates of whey protein isolate, where BLG is the main component [8,14,15,16,17,18,20,24,26,27,28,29,30,31,32,33]. Only recently have the dynamic properties of adsorbed and spread layers of lysozyme fibrils at the liquid–gas interface been studied [25]. In this case, some properties of the layers differed from those of BLG layers as a result of stronger cohesion between the lysozyme aggregates at the interface. To the best of our knowledge, no adsorption or spread layers of fibrils of other animal proteins have been studied at the liquid–gas interface. The interrelationship between the protein structure and the properties of fibril layers at liquid–liquid and liquid–gas interfaces still remain unclear.

This work aims to fill the existing gap in our knowledge and to investigate the adsorption layers of fibrils of another model protein—α-lactalbumin (ALA). ALA is a small protein (14 kDa) that can be found in mammal milk. It consists of 123 amino acid residues organized in two domains and is homologous to lysozyme but characterized by different aggregating properties [34]. ALA is of particular interest because its tertiary structure is not very stable, and at a pH of about 2, the protein adopts a molten globule state without the application of any chemical denaturants or change in temperature. Moreover, its fibrils can refold from one polymorphic state to another through mild changes in external conditions [35].

## 2. Experimental Part

### 2.1. Materials

ALA (Mw ≈ 14 kDa) and thioflavin T (ThT) were purchased from Sigma-Aldrich (Darmstadt, Germany). Sodium chloride from Vekton (Yekaterinburg, Russia) was heated in an oven to 750 °C to remove possible organic impurities. The protein solutions in a phosphate buffer (ionic strength I = 0.020 M) were prepared using triply distilled water and stored in a refrigerator for no longer than three days. The second and third distillations of water were performed using equipment made of glass only. The buffer solution was prepared by mixing solutions of NaH_2_PO_4_ from Sigma Aldrich (Darmstadt, Germany) and Na_2_HPO_4_ from Sigma Aldrich (Darmstadt, Germany). Hydrogen chloride from Vekton (Yekaterinburg, Russia) was used to decrease the solution’s pH.

### 2.2. Sample Preparation

The fibrils were prepared via the heating of a concentrated ALA solution in agreement with a procedure usually used for these aims [34,36]. A 10 wt% ALA solution with pH 2 was dialyzed against a HCl solution of pH 2 using a cellulose membrane (Sigma-Aldrich, Darmstadt, Germany) to eliminate the admixtures of low-molecular weight and inorganic ions. The dialyzed solution with a protein concentration of ~2 wt% was filtered through a membrane with a pore size of 200 nm from Vladipore membranes (Vladimir, Russia) to remove undissolved particles. After that, NaCl was added to the solution up to a concentration of 0.15 M, and the flask with the solution was incubated for 48 h at 60 °C at a flask rotation rate of around 120 rpm. After heating, the solution was cooled by placing the flask into an ice-water bath. The conventional thioflavin T test confirmed the formation of fibrils (see Figure S1 of the Supporting Information). The fluorescence emission spectra were recorded in a wavelength range of 460–650 nm with the excitation at 448 nm using a fluorescence spectrophotometer Fluoromax Plus from Horiba (Kyoto, Japan). The slit widths for excitation and emission were both 1 nm. The mean length of the fibrils exceeded 1 μm, and they had a tendency to stick together (see Figure S2 of the Supporting Information). The increase in the solution pH to 7 through the dilution of the dialyzed solution by the buffer did not lead to visible changes in the fibrils (see Figure S3 of the Supporting Information).

The obtained fibrils were purified via centrifugation (50,000× *g*, about 30 min using a ultracentrifuge Optima L-100XP from Bekman Coulter (Brea, CA, USA)) and through the replacement of the supernatant with water at pH 2. After that, the fibrils were resuspended through shaking (see Figure S2 of the Supporting Information). This purification procedure was repeated twice. The final concentration of the fibril dispersion was determined using UV–Vis spectroscopy using a UV-1800 from Shimadzu (Kyoto, Japan) instrument. The absorption spectra were measured in a wavelength range from 250 to 650 nm for solutions with known concentrations of native ALA (see Figure S1b of the Supporting Information). After that, the spectrum intensities around 280 nm were used to obtain calibration dependence (see Figure S1c of the Supporting Information). The dispersions of ALA fibrils and native ALA solutions have the same spectra, giving us the possibility to determine the protein concentration using the determined calibration line (see Figure S1c of the Supporting Information).

### 2.3. Methods

A computer-controlled Langmuir trough (KSV NIMA, Espoo, Finland) with two moving polytetrafluoroethylene (PTFE) barriers and a Wilhelmy plate made of filter paper was employed for the measurements of the dynamic surface tension and spreading experiments. In the latter case, the protein solution or fibril dispersion were dripped onto a liquid subphase using an inclined glass plate [26]. The Wilhelmy plate was positioned parallel to the barriers in the center of the trough. Measurements of the surface tension with an accuracy of approximately ±0.2 mN/m as a function of the surface area between the barriers were taken only 10 min after the fibrils or the native protein were spread in order to equilibrate the system.

The oscillating barrier method used to measure the dilational dynamic surface elasticity is described in detail elsewhere [37,38]. The symmetric oscillations of the two PTFE barriers gliding back and forth out of phase along the polished brims of the Langmuir trough led to oscillations of the surface area and, consequently, of the surface tension. The oscillation frequency and amplitude were 0.1 Hz and 5%, respectively. The induced oscillations of the surface tension were determined via the Wilhelmy plate method, which was positioned at equal distances to the two moving barriers and perpendicular to the main axis of the trough. The liquid surface area in the Langmuir trough could be changed within a range of 520 to 30.0 cm^2^ [37]:

$$E=E_{r}+iE_{i}=\frac{\delta \gamma }{\delta \ln A}$$

where *δγ* and *δA* are the complex increments of the surface tension and surface area, respectively.

The imaginary part of the dynamic surface elasticity was always much less than the real part for the systems under investigation, and therefore, only the real part is represented and discussed below. The corresponding accuracy is approximately ±3%.

The fibril layers were transferred from an aqueous subphase onto a freshly cleft mica plate using the Langmuir–Schaeffer method and then dried in a desiccator. After that, the micromorphology of the layers was determined via atomic force microscopy (AFM). A NTEGRA Prima instrument (NT-MDT, Moscow, Russia) in a semi-contact regime was used for these purposes.

## 3. Results

### 3.1. Native Protein

The conformations of protein molecules at the liquid–gas interface have been discussed for about a century. Classic studies considered the unfolding of globular proteins in the course of adsorption [39,40], while more recently, the application of neutron reflectometry [41,42,43], Fourier-transform infrared reflection absorption spectroscopy [44,45], and surface dilational rheology [46,47] have not discovered noticeable changes in the protein’s tertiary structure. A similar conclusion was reached regarding the preservation of the globular structure of ALA in the surface layer, following from simultaneous measurements of the dynamic surface tension and the adsorbed amount at the surface of aqueous solutions [48,49]. The results of the given study on the dynamic surface elasticity of ALA solutions also agree with the idea that the protein’s tertiary structure does not change in the course of adsorption.

**Figure 1:**
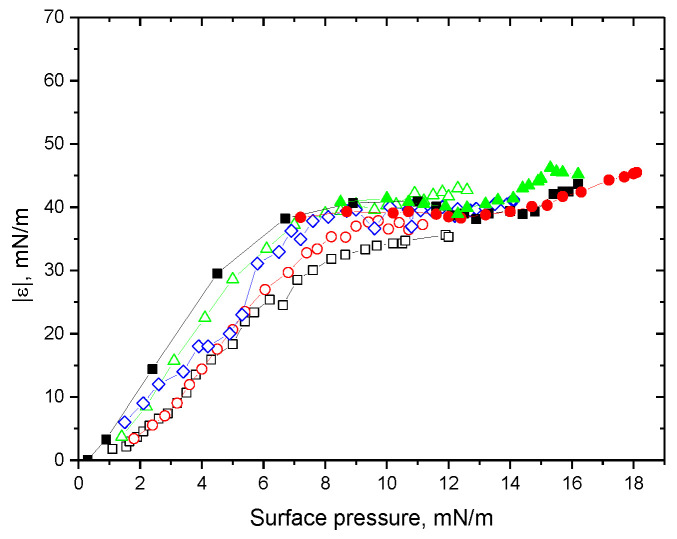
Dependencies of the dynamic surface elasticity on surface pressure of ALA solutions at pH 2 with 0.1 M NaCl at protein concentrations of 0.3 (black squares), 0.5 (red circles), and 1 μM (green triangles); without NaCl at protein concentrations of 1 (black hollow squares), 3 (red hollow circles), 10 (green hollow triangles), and 20 μM (blue hollow diamonds).

**Figure 2:**
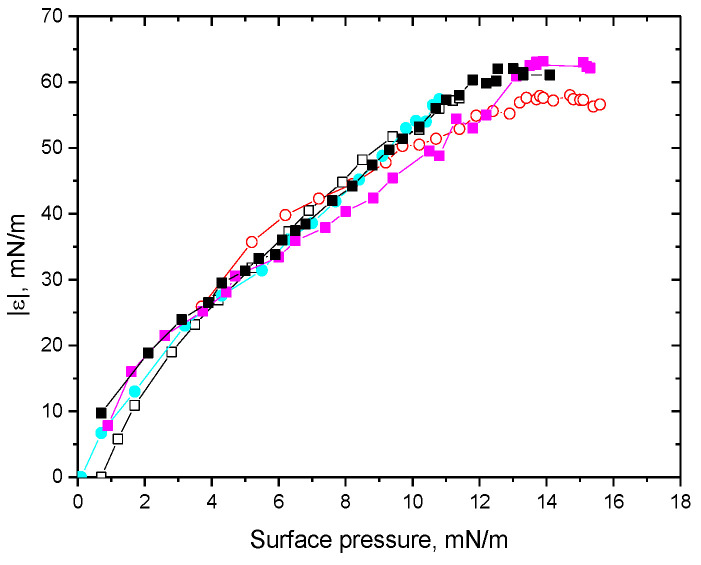
Dependencies of the dynamic surface elasticity on surface pressure of ALA solutions at pH 7 with 0.1 M NaCl at protein concentrations of 0.3 (cyan circles), 0.5 (pink squares), and 1 μM (black squares); without NaCl at protein concentrations of 1 (black hollow squares) and 4 μM (red hollow circles).

All the kinetic dependencies of the dynamic surface elasticity (see Figures S4 and S5 of Supporting Information) and the corresponding dependencies of the surface elasticity on surface pressure (Figure 1 and Figure 2) are monotonic within the investigated concentration range of ALA (10^−7^–10^−5^ M). This behavior indicates that the protein preserves its globular structure in the surface layer [47]. At the same time, the changes in surface properties with the surface age at pH 2 are slightly slower than at pH 7 at similar concentrations due to a slightly lower diffusion coefficient of the molten globules than of the native molecules in the latter case (see Figures S4–S7 of Supporting Information) [48]. The dynamic surface elasticity at pH 2 is noticeably lower than that at pH 7 as a result of a looser adsorption layer of molten globules with a destroyed tertiary structure (Figure 1 and Figure 2) [50].

The increase in the solution’s ionic strength leads to a significant acceleration of the changes of surface properties (see Figures S4–S7 of Supporting Information) but almost does not change the dependencies of the surface elasticity on surface pressure (Figure 1 and Figure 2). This behavior means that the increase in the ionic strength influences only the adsorption kinetics by decreasing the electrostatic adsorption barrier but almost does not change the structure of the adsorption layer. It is possible to observe only a small effect, close to the error limits, at pH 2 when the addition of NaCl leads to a slight increase in the surface elasticity of the layer of molten globules at a given surface pressure (Figure 1). The increase in the solution’s ionic strength at pH 2 leads to stronger changes in the kinetic dependencies of the surface properties than the changes at pH 7, presumably due to the higher value of the globule charge in the former case (see Figures S4–S7 of Supporting Information) [51].

### 3.2. Fibrils of Proteins

**Figure 3:**
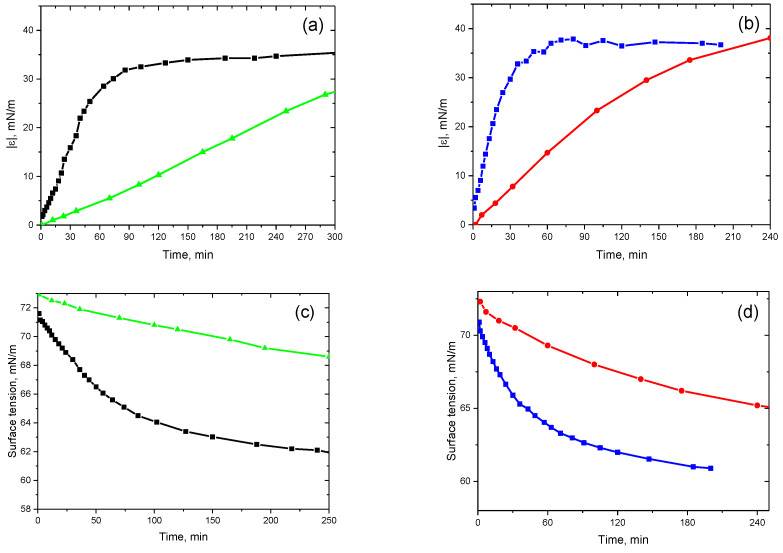
Kinetic dependencies of the dynamic surface elasticity (**a**,**b**) and surface tension (**c**,**d**) for the solutions of native ALA at protein concentrations of 1 µM (black squares) and 3 μM (blue squares) and of ALA fibril dispersions at protein concentrations of 1 µM (green triangles) and 3 μM (red circles) at pH 2.

The dynamic surface properties of dispersions of ALA fibrils at pH 2 changed slower than those of the solutions of the native protein at similar concentrations as a result of the larger particles diffusing to the interface. Note that the fibrils have a lower diffusion coefficient than protein globules (Figure 3a–d). These data indicate a weaker influence of the hydrolyzed peptides of low molecular weight as compared, for example, with dispersions of lysozyme fibrils where the same purification method did not allow the removal of the admixtures, and the surface properties changed faster for fibril dispersions than for solutions of the native protein [25]. At the same time, slow fibril adsorption hampers the measurement of the surface tension values of ALA fibril dispersions in a steady state.

**Figure 4:**
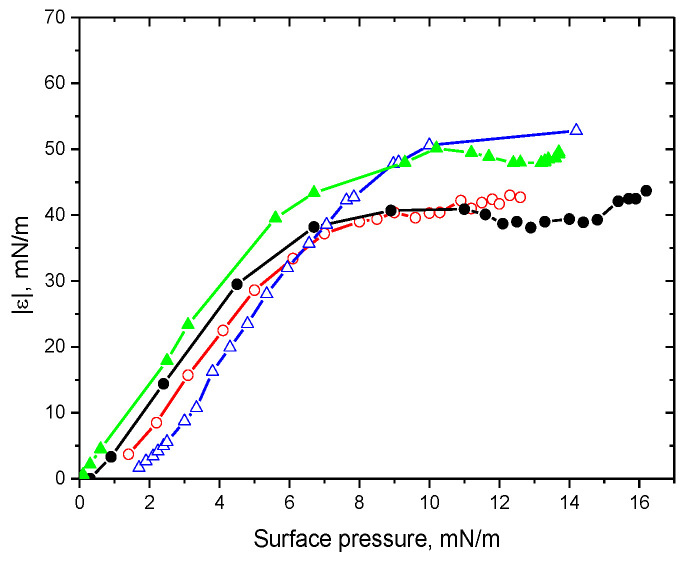
Dependencies of the dynamic surface elasticity on the surface pressure for ALA solutions of native protein with (black circles) and without NaCl (red hollow circles) and ALA fibril dispersions with (green triangles) and without 0.1 M NaCl (blue hollow triangles) at pH 2.

The properties of the adsorption layers of ALA fibril dispersions at pH 2 are presumably determined mainly by fibrils and not by admixtures, unlike in the cases of lysozyme and BLG dispersions. In the latter case, the dependencies of the dynamic surface elasticity on surface pressure coincided with the corresponding results for native protein solutions [26]. On the contrary, the surface elasticity of ALA fibril dispersions was higher than the data for native protein solutions, at least at surface pressures higher than about 7 mN/m (Figure 4). The results for the spread and adsorbed fibril layers almost coincided in the system under investigation, unlike the corresponding data for the fibrils of other proteins [25], thereby corroborating a relatively low concentration of admixtures in the investigated dispersions.

**Figure 5:**
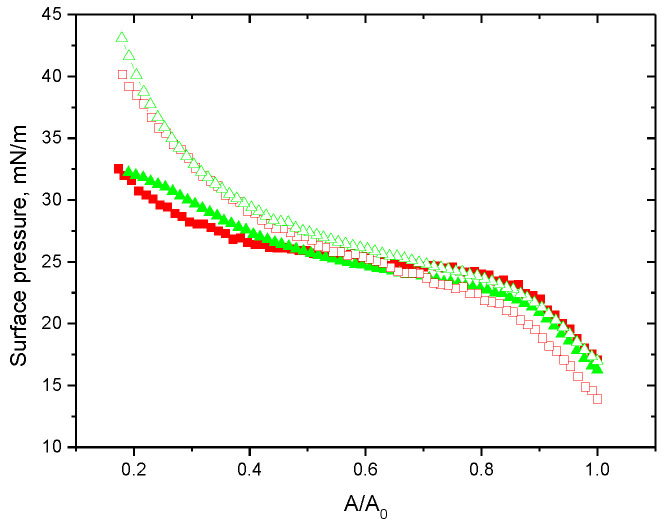
Compression isotherms of adsorbed layers of ALA native protein (red squares) and ALA fibrils without NaCl (red hollow squares), and ALA native protein (green triangles) and ALA fibrils with NaCl (green hollow triangles) at pH 2.

The difference between the adsorption layers of ALA fibrils and the native protein becomes more pronounced upon surface compression. At relatively low changes in the surface area, approximately less than twice, the surface pressure isotherms are similar for the layers of native protein and fibrils, but at higher compressions, when the layer becomes thicker, the surface pressure increases stronger in the latter system (Figure 5). It is possible to assume that the transition of fibrils from the proximal region of the surface layer to the distal region requires higher surface stresses than the formation of a bilayer of protein globules. At lower surface compressions, only some parts of a fibril can be squeezed out from the surface, but stronger compression can separate the whole fibril from the contact with the air phase. The latter event requires higher energy, which can depend on the surface density and rigidity of the fibrils as in the case of a polymer chain detachment from a liquid surface [52].

**Figure 6:**
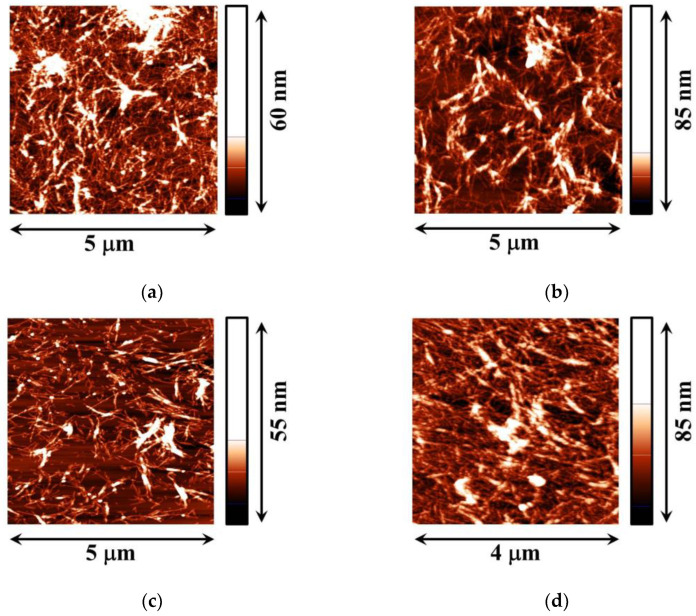
AFM images of the adsorbed layer of ALA fibrils at pH 2 before compression without NaCl (**a**), after compression without NaCl (**b**), before compression with 0.1 M NaCl (**c**), and after compression with 0.1 M NaCl (**d**).

The transfer of the adsorption layer of ALA fibrils onto the surface of mica and its analysis via AFM shows a rather dense layer of fibrils (Figure 6). It is not a monolayer, and some fibrils overlap. Moreover, some ALA fibrils can stick together in the surface layer and form rather dense surface aggregates. This dense surface structure of relatively rigid fibrils provides a higher surface elasticity and higher surface pressure than for the layer of protein globules (Figure 4). The surface compression results in an increase in the fibril surface density (Figure 6). The increase in the ionic strength of ALA fibril dispersions leads to the acceleration of the adsorption kinetics and to faster changes in the surface properties as compared to the case of ALA solutions (see Figures S8 and S9 of the Supporting Information). At the same time, the dependencies of the surface elasticity on surface pressure do not change, indicating that the ionic strength influences only the adsorption kinetics but not the adsorption layer structure. The AFM images confirm this conclusion (Figure 6).

**Figure 7:**
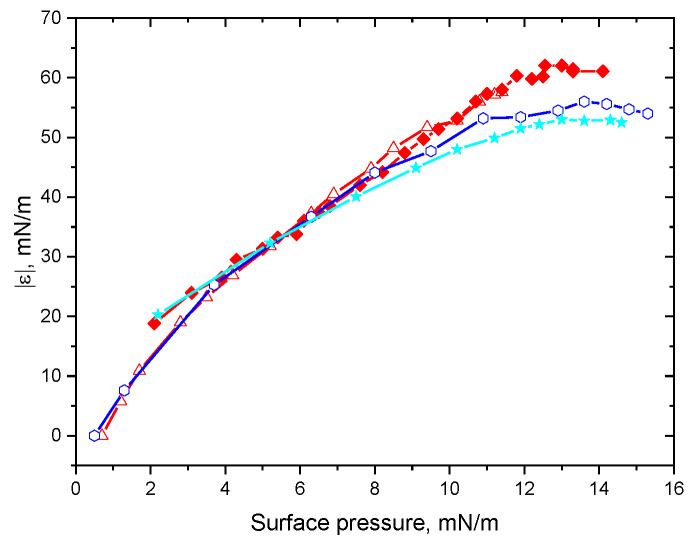
Dependencies of the dynamic surface elasticity on the surface pressure of 1 μM ALA solutions of native protein with (red diamonds) and without 0.1 M NaCl (red hollow triangles) and of ALA fibril dispersions with (cyan asterisks) and without (blue hollow hexagons) 0.1 M NaCl at pH 7.

The increase in the pH of the fibril dispersions leads to different results. Similarly to the case of native ALA solutions, at pH 7, the dynamic surface elasticity is higher than at pH 2. At the same time, the difference between the dynamic surface properties of protein solutions and fibril dispersions almost disappear (Figure 7). The application of AFM shows that this result is connected with a much lower local concentration of fibrils in the surface layer. Although the concentration of fibrils in the bulk phase is fairly high (see Figure S3 of Supporting Information), they are almost not adsorbed at the liquid surface at pH 7. The AFM images of the adsorption layers show only some traces of fibrils in this case (see Figure S10 of Supporting Information). The adsorption layer at pH 7 presumably consists mainly of admixtures of smaller size molecules, which are not visible via AFM. These admixtures could be hydrolyzed peptides or possibly native and partially denatured protein molecules.

**Figure 8:**
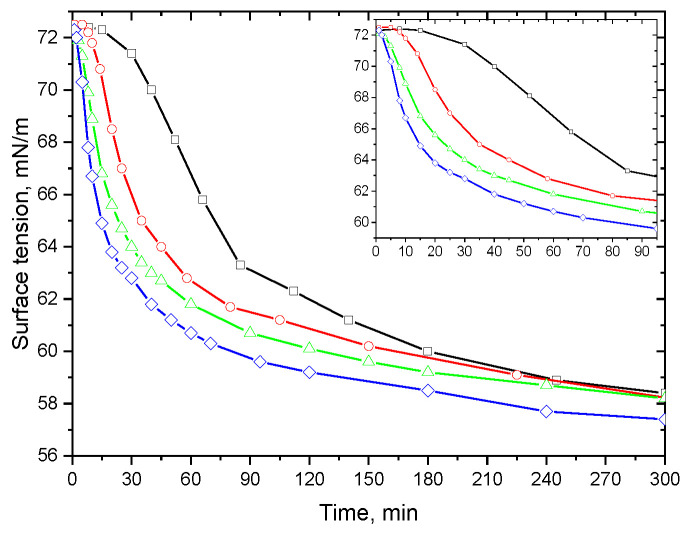
Kinetic dependencies of surface tension of 1 μM ALA fibril dispersions at pH 7 and at various times after the dispersion preparation: 1 h (black squares), 5 h (red circles), 10 h (green triangles), and 24 h (blue diamonds).

The obtained results show that the admixtures are not formed in the surface layer but adsorbed from the bulk phase. Note that the concentration of admixtures is relatively low at pH 2, and they do not significantly influence the surface properties (cf. comments above). Therefore, the formation of admixtures in the bulk phase presumably occurs at the increase in the solution’s pH of up to 7, when the fibrils become unstable. Although the AFM images do not allow the observation of the fibril destruction, presumably due to a slow rate of this process, it leads to changes in the kinetic dependencies of the surface properties as a result of the high surface activity of these admixtures and their fast adsorption (Figure 8 and Figure S11 of the Supporting Information). The changes in surface properties with the surface age become faster over the course of the solution aging at pH 7 for a few hours, indicating an increase in the admixture concentration. The increase in the adsorption rate occurs because the admixtures have a lower molecular weight and hence a higher diffusion coefficient as compared to fibrils. In this case, the adsorption of fibrils is presumably insignificant, and the surface pressure and the surface elasticity increase mainly as a result of the adsorption of the admixtures with high surface activity. On the contrary, the kinetic dependencies of the surface properties at pH 2 do not change over the course of solution aging (see Figure S12 of the Supporting Information). The decrease in the fibril adsorption at pH 7 could be connected with the fast filling of the surface layer of hydrolyzed peptides with high surface activity. Another probable reason is the formation of relatively large fibril aggregates in the bulk phase and, thereby, a decrease in the concentration of free fibrils. The aging of ALA fibril dispersions leads to a phase separation and the sedimentation of the aggregates at pH 7 but not at pH 2.

To the best of our knowledge, the relatively fast destruction of amyloid fibrils over a few hours has not been observed for fibrils of other proteins. The surface properties proved to be highly sensitive to this process for ALA fibrils (Figure 8). Note that Kurouski et al. studied the polymorphism of ALA fibrils and the transitions between two polymorphs as a result of alterations in the solution temperature and ionic strength [35]. Later, these authors also considered the influence of pH on the polymorph transitions but not on the release of small molecules from fibrils [53]. The surface properties of recently studied dispersions of lysozyme and BLG fibrils at pH 7 do not depend on the solutions’ age, presumably due to the higher stability of the fibrils of these two proteins [26].

## 4. Conclusions

The dynamic surface properties of aqueous dispersions of ALA fibrils differ significantly from those of previously studied dispersions of BLG and lysozyme fibrils. The formation of molten globules of ALA at pH 2 results in looser adsorption layers at the solution–air interface with a lower dynamic elasticity than for layers of native protein solutions at pH 7. At the same time, ALA fibrils at pH 2 are relatively stable, do not release hydrolyzed peptides into the dispersion, and can be easily purified from admixtures, unlike the fibrils of other proteins, for example, lysozyme and BLG. The dynamic surface elasticity of ALA fibril dispersions is higher than that of native ALA solutions and the adsorption kinetics is slower. The stability of fibrils and their dispersions are lost at pH 7. The fibrils start to aggregate, leading to a phase separation. On the other hand, they release small molecules (hydrolyzed peptides) into the bulk phase. This effect results in significant changes in the kinetic dependencies of surface properties during the aging of the dispersion and, to the best of our knowledge, has not been observed for the fibrils of other proteins. On the other hand, this has to be taken into account in the preparation of stable emulsions and foams.

## Data Availability

Not applicable.

## Supplementary Materials

The following supporting information can be downloaded at: https://www.mdpi.com/article/10.3390/polym15193970/s1, Figure S1. (a) The fluorescence intensity of native ALA (black squares) and ALA fibrils at pH 2 (blue circles), and at pH 7 (green triangles). Protein concentration is 48 μM and λexc = 448 nm. (b) Spectra of UV absorbance for native ALA solutions (black, red line, and green lines and ALA fibril dispersions (blue line) at pH 2; (c) calibration line based on spectra for solutions of native ALA. Figure S2. AFM images of ALA fibril dispersion in solution bulk phase at pH 2 before (a) and after purification (b). Figure S3. AFM image of ALA fibril dispersion in solution bulk phase at pH 7. Figure S4. Kinetic dependencies of the dynamic surface elasticity of ALA solutions at protein concentrations of 1 (black squares), 3 (red circles), 10 (green triangles), and 20 μM (blue diamonds) at pH 2. Figure S5. Kinetic dependencies of the dynamic surface elasticity of ALA solutions at protein concentrations of 1 (black squares), 4 (red circles), and 5 μM (green triangles) at pH 7. Figure S6. Kinetic dependencies of surface tension of 1 μM ALA solutions with (black circles) and without 0.1 M NaCl (black hollow circles) at pH 2. Figure S7. Kinetic dependencies of surface tension of 1 μM ALA solutions with (black circles) and without 0.1 M NaCl (black open circles) at pH 7. Figure S8. Kinetic dependencies of surface tension of 1 μM solutions of ALA fibrils with (black squares) and without 0.1 M NaCl (red circles) at pH 2. Figure S9. Kinetic dependencies of the dynamic surface elasticity of 1 μM ALA fibril dispersions with (black squares) and without 0.1 M NaCl (red circles) at pH 2. Figure S10. AFM images of the adsorbed layer of ALA fibrils at pH 7 and a protein concentration of 1 μM before (a) and after (b) compression. Figure S11. Kinetic dependencies of the dynamic surface elasticity (a) and dependencies of the surface elasticity on surface pressure (b) of 1 μM ALA fibril dispersions at pH 7 and at various times after the dispersion preparation; 1 h (black squares), 5 h (red circles), 10 h (green triangles), and 24 h (blue diamonds). Figure S12. Kinetic dependencies of surface tension (a) and dynamic surface elasticity (b) of ALA fibril dispersions at a concentration of 3 μM at pH 2 and at various times after the dispersion preparation, i.e., 1 h (red circles), 24 h (green triangles).
