# Supplementary material for: Dynamic Surface Properties of α-Lactalbumin Fibril Dispersions
Source: Polymers (Basel). 2023 Oct 2;15(19):3970. doi: 10.3390/polym15193970 (PMC10574873; doi:10.3390/polym15193970)
Supplement: Supplementary file 1 [file polymers-15-03970-s001.zip › polymers-2525113-supplementary.pdf]

Supporting information

Dynamic Surface Properties of α-lactalbumin Fibril Dispersions

Boris Noskov [1], Giuseppe Loglio [2], Reinhard Miller [3], Olga Milyaeva [1], Maria Panaeva [1] and Alexey Bykov [1,*]

[1] Institute of Chemistry, St. Petersburg State University, Universitetsky pr. 26, St. Petersburg 198504, Russia;

[2] Institute of Condensed Matter Chemistry and Technologies for Energy, 16149 Genoa, Italy;

[3] Department of Physics, Technical University of Darmstadt, 64289 Darmstadt, Germany;

* Correspondence: a.bykov@spbu.ru

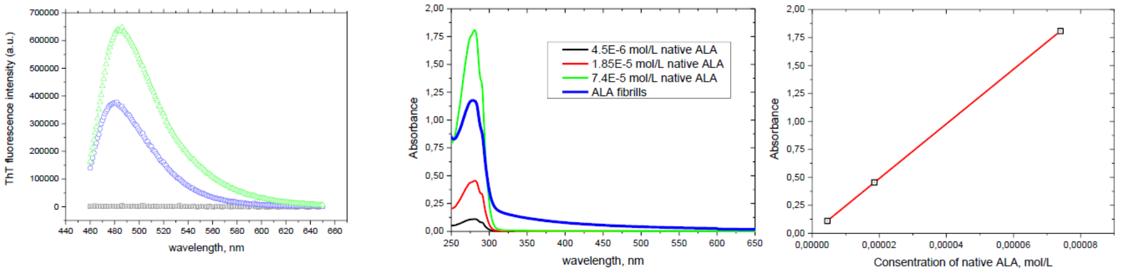

(a) (b) (c)

Figure S1. (a) The fluorescence intensity of native ALA (black squares) and ALA fibrils at pH 2 (blue circles), and at pH 7 (green triangles). Protein concentration is 48 μM and λ_{exc}=448 nm. (b) Spectra of UV-absorbance for native ALA solutions (black, red line and green lines) and ALA fibril dispersions (blue line) at pH 2.; (c) Calibration line based on spectra for solutions of native ALA.

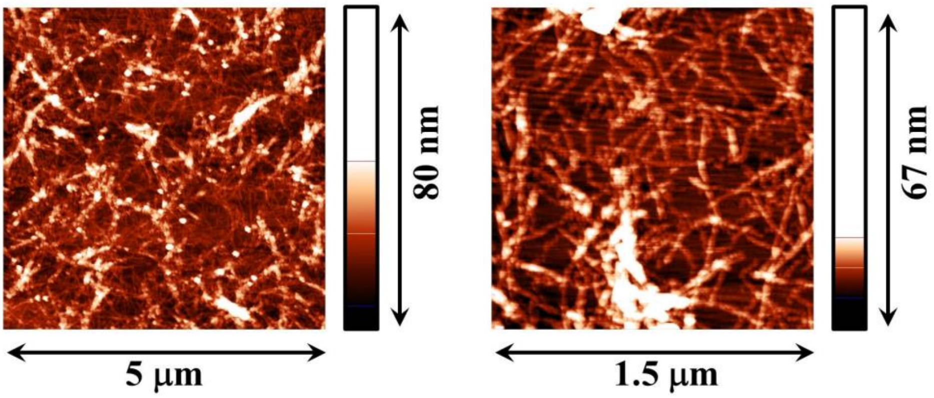

(a) (b)

Figure S2. AFM images of ALA fibril dispersions at pH 2 before (a) and after purification (b).

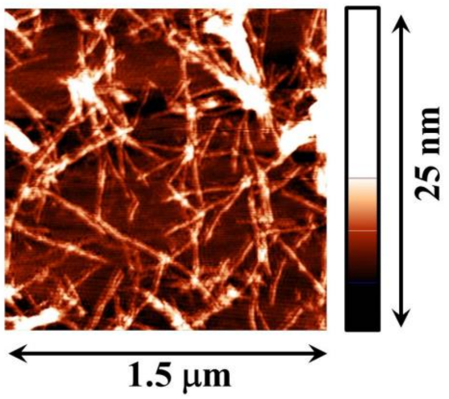

Figure S3. AFM image of an ALA fibril dispersion at pH 7

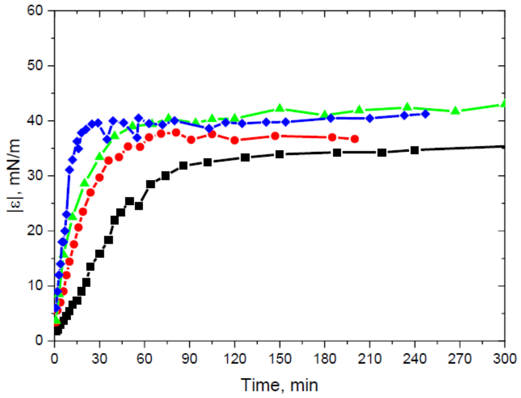

Figure S4. Kinetic dependencies of the dynamic surface elasticity of ALA solutions at protein concentrations of 1 (black squares), 3 (red circles), 10 (green triangles) and 20 μM (blue diamonds) at pH 2.

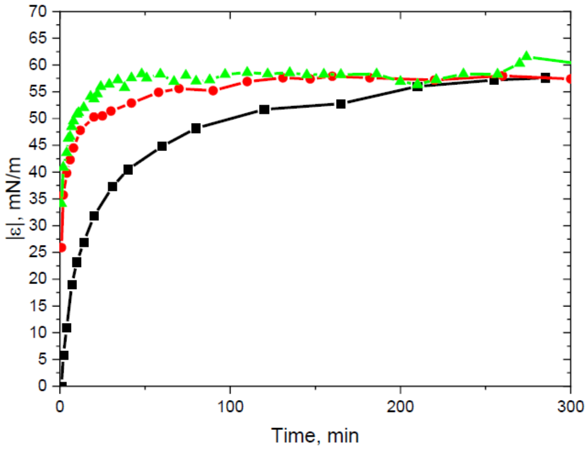

Figure S5. Kinetic dependencies of the dynamic surface elasticity of ALA solutions at protein concentrations of 1 (black squares), 4 (red circles) and 5 μM (green triangles) at pH 7.

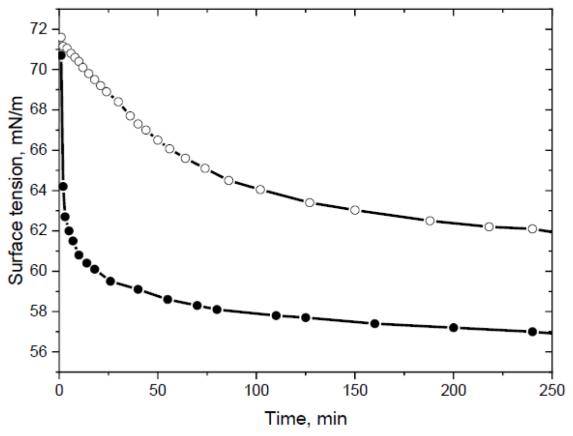

Figure S6. Kinetic dependencies of surface tension of a 1 μM ALA solution with (black circles) and without 0.1 M NaCl (black open circles) at pH 2.

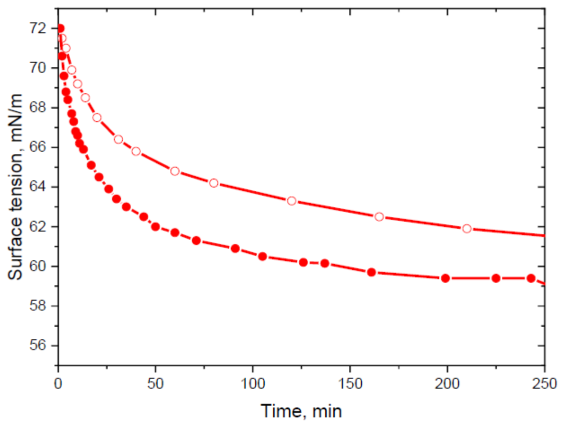

Figure S7. Kinetic dependencies of surface tension of a 1 μM ALA solution with (black circles) and without 0.1 M NaCl (black open circles) at pH 7.

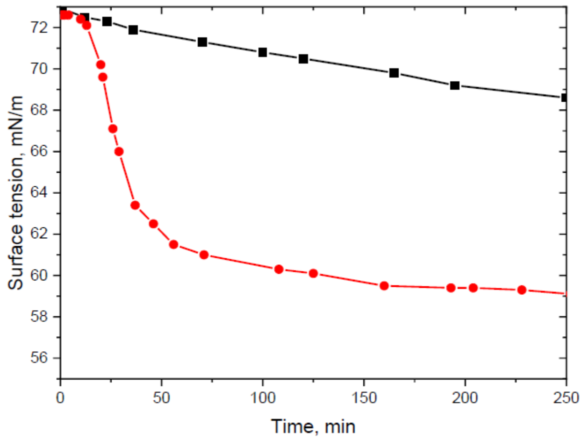

Figure S8. Kinetic dependencies of surface tension of a 1 μM solution of ALA fibrils with (black squares) and without 0.1 M NaCl (red circles) at pH 2.

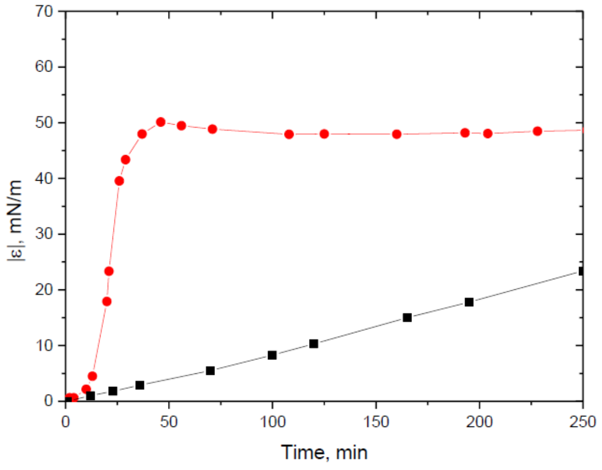

Figure S9. Kinetic dependencies of the dynamic surface elasticity of a 1 μM ALA fibril dispersion with (black squares) and without 0.1 M NaCl (red circles) at pH 2.

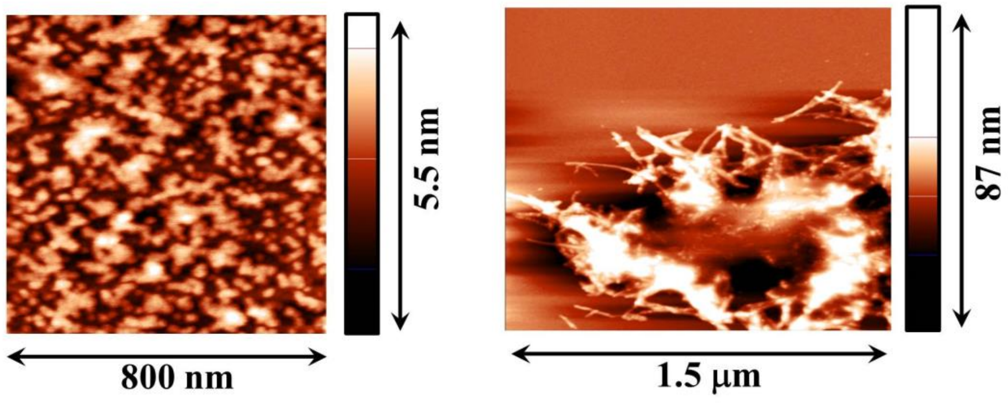

(a) (b)

Figure S10. AFM images of the adsorbed layer of ALA fibrils at pH 7 and a protein concentration of 1 μM before (a) and after (b) compression.

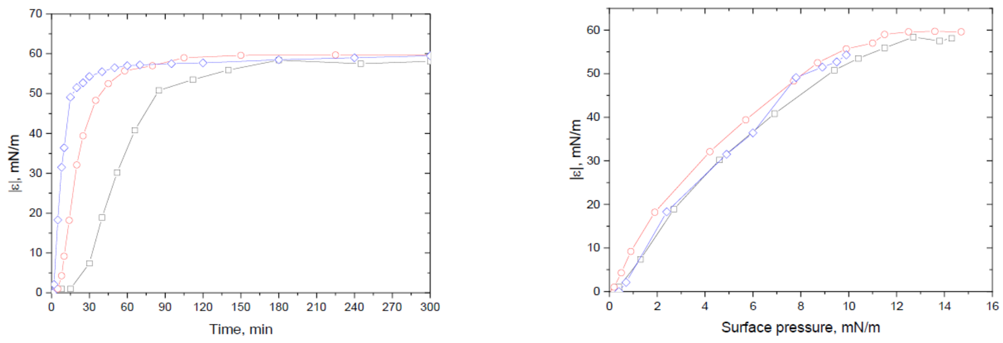

Figure S11. Kinetic dependencies of the dynamic surface elasticity (a) and dependencies of the surface elasticity on surface pressure (b) of a 1 μM ALA fibril dispersion at pH 7 and at various times after the dispersion preparation: 1 hour (black squares), 5 hours (red circles), 10 hours (green triangles), 24 hours (blue diamonds).

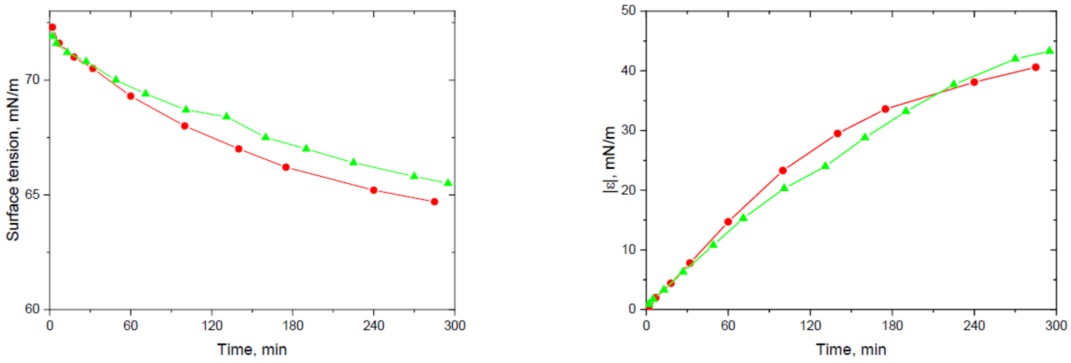

(a) (b)

Figure S12. Kinetic dependencies of surface tension (a) and dynamic surface elasticity (b) of an ALA fibril dispersion at a concentration of 3 μM at pH 2 and at different times after the dispersion preparation: 1 hour (red circles), 24 hours (green triangles).
